# Facilitators and barriers of herbal medicine use in Accra, Ghana: an inductive exploratory study

**DOI:** 10.1186/s12906-016-1124-y

**Published:** 2016-05-26

**Authors:** Lydia Aziato, Hannah Ohemeng Antwi

**Affiliations:** Department of Adult Health, School of Nursing, University of Ghana, Legon, Accra Ghana; School of Nursing, College of Health Sciences, University of Ghana, P.O. Box LG 43, Legon, Accra Ghana

**Keywords:** Qualitative, Complementary and alternative medicine (CAM), Herbal clinic, Effectiveness

## Abstract

**Background:**

The use of complementary and alternative medicine including herbal medicine is increasing in many countries including Ghana. However, there is paucity of research on the perspectives of patrons of herbal medicine regarding the facilitators and barriers of herbal medicine use. This study sought to investigate the facilitators and barriers of herbal medicine among Ghanaian adults who use one form of herbal medicine or the other.

**Methods:**

The study employed an inductive exploratory qualitative approach. It was conducted at a private herbal clinic in Accra. Purposive sampling was employed to recruit 16 participants. Data collection was through individual face-to-face interviews and these were transcribed and analysed using content analysis procedures.

**Results:**

It was realized that the factors that enhanced the use of herbal medicine included use of convincing information to enhance the initiation of herbal medicine use, effectiveness of herbal medicine, personal preference for herbal medicine, perceived ineffectiveness of western medicine and integration of spirituality in herbal medicine. The factors that hindered herbal medicine use included negative perceptions and attitudes about herbal medicine, poor vending environment, poor knowledge of vendors, high cost of herbal products at credible herbal clinics and inconsistent effectiveness of some herbal products. Participants desired that the national health insurance scheme will cover the cost of herbal medicine to alleviate the financial burden associated with herbal medicine use.

**Conclusion:**

Although some Ghanaians patronize herbal medicine, the negative perceptions about herbal medicine resulting from deceitful producers and vendors call for enhanced education and monitoring to ensure that effective herbal products are used.

## Background

Complementary and alternative medicine (CAM) involves the use of non-conventional approaches in health care such as herbal medicine, acupuncture, hypnosis, music, and prayer [1, 2]. The use of herbal medicine in health care is gaining a lot of popularity in contemporary health systems and it is being promoted using different marketing strategies such as advertisement on the radio, newspaper, television and the internet [3, 4]. Complementary and alternative medicine may be used in conjunction with conventional/orthodox/western medicine or as a single therapy [5]. The use of herbal medicine may be initiated through the recommendation of health professionals, other lay people or through a personal preference [6]. Globally, about 80 % of the populace use one form of herbal medicine or the other [7]. However, the number of people who use herbal medicine may vary from urban and rural settings although both rural and urban dwellers use herbal medicine [8, 9]. However both the uneducated and the educated use herbal medicine including health professionals and university professors [10].

The use of herbal medicine for various diseases dates back to the ancient man where there was no western medicine. The processing and formulations of herbal medicine have improved over the years with sophisticated machines used in the production and packaging of herbal medicine [11–13]. Thus, herbal medicine is available currently in various forms including tablets, capsules, ointment, decoctions, powders and tincture liquids [14, 15]. There are scientific establishments dedicated to investigating herbal medicine where the active ingredients are isolated and analysed to enhance the safety of herbal medicine [16]. Regulatory bodies responsible for food and drugs have responsibility to ensure the safety of herbal products used by individuals [1, 17]. It means that herbal medicine producers are to be licensed and their products investigated and certified before they are sold to the public [14, 16, 18]. In Ghana, herbal medicine producers are licensed by the Food and Drug Authority and the Centre for Research into Plant Medicine investigates the drug for efficacy and safety before the licensing. Training of herbal medicine practitioners has been instituted in many countries including Ghana to ensure that providers are well informed about specific herbal medicines and its use [17]. Over the years, herbal medicine providers were not educated and their training was informal and some were trained by their family members who were herbalist [2]. Therefore, the inclusion of herbal medicine providers in the regular education system even at the tertiary level goes to increase the credibility of herbal medicine in contemporary healthcare as occurs at the Kwame Nkrumah University of Science and Technology (KNUST) in Ghana. The training is a 4 year Bachelor of Science (BSc) programme. Upon completion, graduates are licensed by the Traditional and Alternative Medicine Council as herbal medicine practitioners and they consult and prescribe herbal medicine for clients both in the government and private hospitals in Ghana.

Various parts of plants and tress such as the leaves, roots, fruits and bark of stems are used to prepare herbal medicine [8, 19–21]. The plant parts can be boiled, dried or pounded/ground before use either in the raw state or used in mechanically processed products [22] for many health problems such as Infertility [20, 23], Dysmenorrhoea [9, 24], Fibroid [2, 25], Migraine [5], Diabetes [26], Malaria [23], Haemorrhoids [27], Rheumatoid Arthritis [28] and Hepatitis [9]. Various effectiveness of herbal medicine for these health problems have been reported [29, 30]. Many of these studies are based on self-report of participants rather than randomized control trials. However, a few studies report the efficacy of herbal products such as 60 % success for infertility treatment [31]. It is therefore necessary for patients to be given the opportunity to choose the treatment option preferred; and, to afford an informed choice, the positive and negative consequences of the treatment should be emphasized. Doctors and nurses should respect that choice and support the patient when using herbal medicine.

The reasons why people use herbal medicine included the natural nature of herbal medicine [32], no or minimal side-effect [33], delay at the hospital [1], ineffective western medicine, having control over treatment decisions, easy accessibility and availability and lack of faith in conventional medicine [3, 6, 34]. Conversely, there are some factors that discourage some people from using herbal medicine such as inappropriate dosage, uninformed providers, poor packaging and labelling, fake products, fear of dangerous effect and unreliable herbal service vendors and practitioners [6, 8]. Some negative effects of herbal medicine impair the skin, eye, liver, kidney and the gastro-intestinal tract [30, 35, 36]. This indicates that the perception that herbal medicine has no side-effects is unfounded. Therefore we suggest that clients who adversely react to herbal medicine should not use it.

Although negative factors are associated with herbal medicine, many people continue to use herbal medicine. The factors influencing herbal medicine use have been investigated in many countries; but, there is paucity of research on the facilitators and barriers of herbal medicine use from the perspectives of users of herbal medicine in Ghana. Therefore this study sought to gain an in-depth understanding of the positive and negative factors that affect herbal medicine use in Ghana.

## Methods

### Design

The study employed an exploratory qualitative approach to investigate the facilitators and barriers of herbal medicine in Ghana. The qualitative approach enabled the researchers to follow-up on emerging reasons why participants used or did not use herbal medicine.

### Setting

The study was conducted at a private herbal clinic in Accra, Ghana. The clinic was commissioned in 1996 and runs out-patient services for clients seeking various herbal products. The average attendance per week is 80–100.

### Target population and sampling technique

The study targeted Ghanaian adults 18 years and above who could speak English, Twi, Ewe or Ga and had the mental capacity to share their thoughts on facilitators and barriers of herbal medicine. Purposive sampling was employed where all clients who met the inclusion criteria were approached and those who consented were recruited until data saturation (no new information emerging from data) was achieved with 16 participants.

### Method of data collection

The first author obtained permission from the leadership of the herbal clinic after she had explained the study to them. She was then assigned a room in the clinic for the interviews. A nurse at the reception served as the recruitment agent and she explained the study to the clients who visited the clinic and those who agreed to participate were referred to the researcher for the interviews. The participants were further screened to confirm their suitability for the study after which they confirmed their readiness to participate in the study. The participants were given an option to re-schedule their meeting; however, they all agreed to be interviewed after they had received the needed care at the clinic. Therefore, participating in the study did not affect the services they received in the clinic.

Individual face-to-face interviews were conducted in English and Twi and audio-taped with a digital voice recorder. The interviews conducted in English were transcribed verbatim and those conducted in Twi were transcribed in English based on the meaning of the statements. The Twi transcripts were discussed with an expert in the Twi language and confidentiality was ensured in the process. The interviewer is experienced in qualitative interviewing and asked open ended questions and used probes as necessary. Participants were told that there were no right or wrong answers and they freely expressed their views. The researchers’ stance that herbal medicine has beneficial effects and patients should be allowed to make their preferred choice of treatment did not influence the data collection process. Interviews lasted between 25 to 35 min per participant.

### Data management and analysis

Concurrent data analysis was undertaken and emerging themes were investigated as the study progressed. The transcripts were checked for accuracy and read several times to fully understand the world of the participants. The data were coded and similar codes were grouped to form themes and sub-themes. This process of data analysis is consistent with the principles of content analysis [37]. The transcripts were then exported to the NVivo software version 10 and the software was used to manage the data.

### Ethical considerations

Ethical clearance was obtained from the Institutional Review Board of the Noguchi Memorial Institute of Medical Research, University of Ghana, Legon. The study was conducted as part of a wider on-going labour pain and religiosity research to gain full understanding on issues of herbal medicine because participants reported that they used herbal medicine. Individual informed consent was obtained from all the participants and voluntary participation were ensured. Anonymity and confidentiality were observed as such, participants were given identification codes such as HMM and HMF with figures 1, 2 and 3, etc. based on participants’ chronologic enrolment into the study.

### Rigour of the study

The study included only participants who sought treatment at a herbal clinic and had experience in the use of herbal medicine. Concurrent analysis enhanced member-checking to follow-up of emerging themes. An inductive process was employed in this study that gave voice to participants’ thoughts on herbal medicine. Field notes and reflexive notes were kept and that gave adequate context to the data and presented data devoid of participants’ ideas. An audit trail was kept to ensure verification of study processes as necessary.

## Results

### Characteristics of study participants

The study participants were 16 made up of 7 females and 9 males. Fourteen participants were Christians, one was a Muslim and one was a traditionalist. Twelve of the participants were married, three were single and one was separated. One participant was a first time user of herbal medicine, one used herbal medicine occasionally and the rest had used herbal medicine for a period ranging from 4 months to over 10 years.

Two major themes: facilitators of herbal medicine use and barriers of herbal medicine use were identified with their corresponding sub-themes.

### Facilitators of herbal medicine use

This theme describes the factors that enhanced the use of herbal medicine. It was realized that initiation of herbal medicine use, effectiveness of the herbal medicine, individual preferences, ineffective western medicine and integration of spirituality in herbal medicine facilitated the use of herbal medicine among participants in this study. The sub-themes for these factors are described with verbatim quotes.

### Initiation of herbal medicine use

Convincing information that led to the initiation of herbal medicine use was acquired through recommendations of friends, family members and the media. The information that influenced participants to use herbal medicine was linked to the effectiveness of herbal medicine.

*‘My sister suggested that I use the herbal medicine. She experienced fibroids and was healed with herbal medicine so that is what encouraged me to use the herbal medicine but I don’t know the specific herb’* (HMF3); *‘a friend of mine encouraged me to use herbal medicine before and I decided to use it’* (HMF6).

*‘I heard about herbal medicine on the radio and TV* (television) *and also people have been talking about it that herbal medicine is good; so, I decided to test it for my problem’* (HMF7).

Information on initiation of herbal medicine was also acquired from information vans of herbal medicine companies.

‘*The herbal clinics have cars that go around and even if you are walking around you will hear them making the announcement about the various herbal medicines they have and how good they are; so, I decided to try’* (HMF6); ‘*they were making announcement about the herbal medicine in a car so much that I decided to buy it from the car’* (HMM4).

Some participants were introduced to herbal medicine when they were young and they continued to use herbal medicine during adulthood.

*‘My grandmother introduced me to herbal medicine because she was a herbalist and she used to give me some when I was a child and when I grew up, I continued to use it’* (HMM8).

However, a few of the participants believed that there was inadequate advertisement on herbal medicine because it is expensive to advertise. As a result, patronage of herbal products is low.

*‘Advertisement on herbal products is low because the adverts are expensive; so, most people do not attend the herbal clinic’* (HMM7).

#### Effectiveness of herbal medicine

Participants attested that herbal medicine was effective and that it had no side-effects. Some were of the view that herbal medicine cures diseases whereas western medicine would only manage the disease.

*‘Oh herbal medicine is good. What I know is that herbal medicine is able to cure the diseases whereas western medicine only manages the diseases but does not cure it. I have experienced that’* (HMF7); *‘As for the herbal medicine, there is nothing like side effects. That is why I like using herbal medicine’* (HMF3).

Some participants confirmed that herbal medicine cured diseases like dysmenorrhoea, jaundice and typhoid fever.

*‘After I started taking herbal medicine, I do not feel any pain when I start menstruating; I am now okay and I am happy; I used to suffer a lot! At first I did not believe herbal medicine works but now, I know it works’* (HMF1).

*‘My dad used herbal medicine for jaundice some 10 years or more back and it worked for him’* (HMF2); *‘I was given herbal medicine that cured my typhoid recently ago so I gained confidence in using herbal medicine after that’* (HMM2).

Some participants believed that herbal medicine cures arthritis, hepatitis, migraine and haemorrhoids.

*‘Herbal medicines cure diseases like Arthritis, Hepatitis and Migraine. Some people with bleeding piles testify that they are healed when they use herbal medicine’* (HMM7); *‘It cures my high BP* (blood pressures) *and Malaria’* (HMMF2).

Although herbal medicine was believed to be effective, some participants were of the view that the user should pray on the medicine to ensure its effectiveness. It was perceived that the herbal medicine may not work if prayer is not added.

*‘You have to pray on the herbal medicine before you use it. Speak whatever you want to see the medicine do for you on it; just normal church prayers that we say, I always pray on my medicine and it works perfectly’* (HMF4).

*‘Some of the herbal drugs may be good but will not work for you. So before I take the herbal medicine, I pray over it. I tell God that the medicine was prepared by human beings so his spirit should enter the drug so that when I take it, it will heal me*’ (HMF5).

### Personal preference for herbal medicine

Some participants used herbal medicine because they *‘loved’* it and they did not like to go to the hospital for western treatment. *‘I love herbal medicine, I love it so much, and it is so good for me. I feel fine when I take it. …I just like herbal medicine more than even Paracetamol’* (HMM1). Others preferred herbal medicine because they hated going to the hospital. *‘Oh, I do not like going to the hospital that is why I like herbal medicine’* (HMM4). Some preferred all forms of herbal medicine preparations be it capsules and syrup administered at the herbal clinic rather than those sold in cars or by the road side.

*‘I like any of herbal medicine either capsules or syrup once I will get well, I will take it. Normally I do not like herbal medicine sold at the road side or those sold in public transport’* (HMF7).

A participant wished that Africans or Ghanaians will use more herbal medicine than western medicine. *‘I will wish that Africans and Ghanaians for that matter will stop using the western medicine and focus more on herbal drugs as they are good’* (HMM6). Some preferred herbal medicine even if it is bitter. *‘I like herbal medicine even if it is bitter; but some people complain about the bitterness. To me, although it is bitter, it does not have side effects and it will make me better so I like it’* (HMM8). Other participants also admitted that herbal medicine was bitter but they managed to drink it because they felt it will help them get better. *‘It is very bitter… I just manage to drink it since I am confident that it will solve my problem and the bitterness will not last forever’* (HMM1).

### Perceived ineffectiveness of western medicine

Herbal medicine was used because of the perceived ineffectiveness of western medicine. Participants were of the view that some western medications have temporary effect and after sometime, the problem re-surfaces.

*‘The tablet they gave me at the hospital for my menstrual pain just made me feel dizzy and I slept and did not feel the pain but when I woke up the pain was still there; it happens every month when I take the tablet’* (HMF1).

*‘Immediately you take it, it will stop and in about a year, it will come back again’* (HMF6).

Some took the hospital medications for several years and had no relieve for their problems. *‘I have taken the hospital medicine several, several, several times. It hasn’t worked for me’* (HMF6). Others felt their body processes did not respond appropriately with western medicine. *‘Sometimes when I visit the hospital, they give me some tablets but it seems it does not work well with my body’* (HMM8). Some participants tried western medicine with over the counter drugs and when they did not get better, they took herbal medicine as an alternative treatment.

*‘I fell sick four days ago; my head was aching and I went to the pharmacy for some pain killers but it has not stopped so I came for herbal medicine’* (HMM9).

Some participants used herbal medicine because they felt that when they go to the hospital, they will wait for a long time to be attended to. *‘I cannot go to the hospital and sit at one place for a long time before I am attended to; at the herbal clinic, I don’t waste too much time’* (HMM9).

In view of the perceived ineffectiveness of the western medicine, some participants took both western and herbal medicine for a particular health problem such as Diabetes. *‘At the moment I am combining the western medicine and the herbal for my sugar problem’* (HMM8).

### Barriers to herbal medicine use

This theme describes factors that were perceived to hinder the use of herbal medicine such as negative perceptions and attitudes, poor vending environment, poor knowledge of vendors, high cost of herbal products at credible herbal clinics and inconsistent effectiveness of some herbal products. The sub-themes generated are described.

### Negative perceptions and attitudes about herbal medicine

Participants were of the view that others see herbal medicine as dangerous and they attach superstition to herbal medicine. *‘Some people say herbal medicine is dangerous so they don’t take it’* (HMF1). *‘when you are staying with somebody and you boil herbal medicine, the person will think that you are coming to do something evil that can affect him/her’* (HMM1).

The mind-set that herbal medicine is bitter also prevent some people from using it. *‘My children will not use herbal medicine because it is their mind-set that herbs are bitter; so, they will never use it even if it is from the right source’* (HMF2).

### Inappropriate vending environment

Participants thought a major barrier to herbal medicine use is the sale of different herbal products in vehicles which may be stationary, mobile or those on a journey. Some also sell in kiosks and on table beside the road. These inappropriate sale outlets did not provide facilities for assessment or privacy for thorough history taking.

*‘Herbal medicine sold at the roadside kiosks, and cars are very dangerous because they do not check your body well to know what is wrong with you. They don’t even ask you questions about your problem’* (HMF1).

*‘…as for roadside herbal medicine and those sold in cars, minus me and my family! I will never buy from those sources’* (HMF2).

Some vendors at these outlets may not be traced after sale of herbal products especially if the products are not good. *‘When you want to locate those you bought the herbal medicine from in cases of challenges with the drug, they will be nowhere to be found’* (HMF7). Some of the drugs from these indecent sources are fake. *‘Sometimes when you take the herbal medicine from cars, it is just like water and you regret buying it’* (HMM9).

### Ignorant and deceitful vendors

In addition, other people fear they will die when they take herbal medicine especially when the vendor is not well informed about the product. *‘The sellers do not have an idea about the use of the herbal medicine and they will give you the wrong information. After taking the medicine, you may end up dying’* (HMF3).

The vendor at the inappropriate sale point such as the roadside and in cars are inadequately informed about herbal medicine and purchasing the drugs from them can be dangerous. It is better to buy herbal products from credible sources.

*‘Those at the roadside are just selling the herbal products and they do not know much about it. So, if you buy from them, it can cause harm to your body; …some of those who go around selling herbal products are also not correct. But those who know and have had experience and have researched the drugs are in a better position to inform you about herbal medicine’* (HMF3).

The vendors at the road side may take too much money for herbal products. They could also sell one herbal drug for many diseases. Such deceitful vendors were believed to deter people from using authentic herbal products.

*‘Those by the road side may give you the wrong drug for a high price and squander your money’* (HMF7).

*‘The uneducated vendors can give you one herbal medicine that cures many diseases like Malaria, Typhoid, Syphilis Gonorrhoea and many more. If you hear such a thing, know that it is a lie. They are discrediting herbal medicine in this country’* (HMF1).

### Variable effectiveness of herbal medicine

Some participants believed that some herbal products are not effective and some had previously used some herbal products that did not have solutions to their problems. *‘When I used the herbal medicine in the past and I did not find the solution to the problem, I stopped; and now, I want to try again’* (HMF4). *‘I used some other herbal medicine but it did not help me and a friend suggested another that I am using now which is better. At first, I got discouraged using herbal medicine’* (HMF6). Some participants thought that they would return herbal products that were not effective to the vendors so that they could be given another product.

*‘If the medicine that they give me does not work for me, I will bring it back so that they may give me another one after checking my body again’* (HMF7).

Some believed that if a particular herbal medicine did not work for them, then it was not meant to be.

*‘Some of the herbal medicines are good for me and others do not work for me; those that do not work for me, I think they are not meant for me’* (HMM3)

### High cost of herbal medicine

High cost of herbal products also deterred people from using herbal medicine. Patrons of herbal medicine were of the view that the cost of herbal products should be reduced so that many more people can afford rather than go to pharmacy shops where unqualified people attend to them.

*‘I think that the cost should be a bit low to encourage a lot of people to use herbal medicine because there are people out there who would wish to come to the Herbal Clinic but because of the cost, they will visit the pharmacy shop instead and sometimes unqualified personnel there just dish out medicines that may cause harm’* (HMF2)

Some felt that the cost of herbal medicine at a credible herbal clinic is too exorbitant. *‘The money they were charging me was very high; so, I only come to the herbal clinic when I can afford’* (HMF3). Some were of the view that the processing and packaging of the herbal medicine contributes to the cost of the herbal product. *‘All the procedures and processes that the herbal drug passes through to become capsules or bottled and labelled so that it will resemble the western medicine, also contributes to the cost of the medicine’* (HMF6).

Some of the participants could not purchase all the herbal drugs that were prescribed. *‘I do not have enough money so I bought only a day’s dose so that later when I get money, I will buy the rest’* (HMM1). It was noted that health insurance did not cover herbal medicine and this further prevents those who cannot afford from using herbal medicine.

*‘Health insurance does not cover herbal medicine and this makes it difficult for some people who cannot afford to use herbal medicine’* (HMM7).

Another participant bought half of the herbal drug prescribed and also lamented over the health insurance.

*‘I bought only half of the drug and explained to them that I do not have enough money on me so I will use it and if I am done I will come and buy the remaining half. Well if the health insurance should cover, it would help’* (HMF7).

A participant was of the view that although herbal products are expensive, once it leads to healing, it is worth it *‘Herbal medicine is expensive but when you know you are paying something that is worth your healing, then you sacrifice for it’* (HMF5)

## Discussion

This study gained a detailed explanation of factors that enhance herbal medicine use. It was realized that individuals start using herbal medicine upon recommendation from those who have personal experiences of its effectiveness and from advertisement. Personal preference for herbal medicine because it was natural and fear of side effects associated with western medicine facilitated herbal medicine use. The barriers of herbal medicine use included herbal medicine being dangerous and bitter, poor environment of vendor, inadequate knowledge of the vendor and high cost of herbal medicine from credible sources. Although the study was conducted at a private herbal clinic, the participants had varied backgrounds and shared their perspectives freely leading to in-depth findings on barriers and facilitators of herbal medicine use. The participants’ views in this study did not indicate a pre-disposition of only positive impressions about herbal medicine. Back translation of interviews were not conducted due to financial constraints. Thus, there could be some degree of inaccuracy in translation in this study although the transcripts were discussed with experts of the local languages.

The study employed an inductive qualitative process through individual interviews. The patrons of herbal medicine shared their thoughts on both the positive and negative factors that influenced herbal medicine. The participants of this study were drawn from a private herbal clinic and this could account for the perception that herbal medicine was expensive. It is possible that those who buy herbal medicine from cars and other retail points may not hold this perception. Again, as for qualitative studies generally, the sample size is small and findings should not be generalized for the entire Ghanaian population of herbal medicine users.

Some of the findings of this study are consistent with previous findings such as the source and content of information that help individuals to initiate herbal medicine use. The information from friends, family members and the media [4, 10] which stressed on the effectiveness of herbal medicine was important. In this regard, although the information was on effectiveness of the herbal medicine, the specific active ingredient of the herb was not known and perhaps the same herb may not be effective for another person. This could account for the variable effectiveness reported [29, 30]. Advertisement on herbal medicine should include the scientific aspect of herbal medicine so that individuals will be well informed about their choice of herbal products. Inadequate advertisement on credible sources of herbal medicine could lead to individuals procuring herbal drugs from unauthorized sources predisposing them to complications [8, 38].

Previous authors support the natural nature of herbal medicine which appeals to users [32]. The natural nature was linked to herbal medicine having minimal or no side-effects [33]. In contrast, participants held that herbal medicine can be dangerous if it is not given by a qualified person or it is a fake product [8]. Therefore, although herbal medicine is natural, users should be cognizant that there could be side effects on the skin, eye, liver, kidney and cause problems like diarrhoea and vomiting [30, 35, 36]. Herbal medicine users should be mindful of these side-effects rather than concentrate on the natural nature of herbal medicine and the perception that it does not cause side-effects. Those who sell western medicine should give the right information about the drugs they sell so that individuals will be well informed about both western and herbal medicines. Some herbs are naturally bitter and this discourages some people from using it [20, 39]. Others were of the view that the natural taste of the herbal drug could alert them of the genuineness of the product. This pre-supposes that herbal medicine producers should not adulterate the natural taste of their produce. The bitter taste did not deter participants since they were more concerned about the therapeutic effect that would cure their health problems.

The perception that herbal medicine gave a permanent solution to a health problem was debatable moreso because some used both products concurrently [40]. Herbal medicine was effective for some participants with dysmenorrhoea and this projected the view that western medicine gives temporary relief. This may not be entirely true because western medicine has also been tried and tested for most health problems [41]. Some participants also reported the variable effectiveness of herbal medicine [29]. Therefore individuals seeking health care with herbal or western medicines should bear in mind that any of these drugs could be effective or ineffective for one reason or the other such as physiological and metabolic differences which may have nothing to do with the potency of the drug. As such, combining herbal and western medicine for a health problem is not recommended and may result in dangerous drug-drug interactions [6]. Thus, information should be available to the public outlining when it is more appropriate to use Western medicine treatment for specific diseases such as HIV/AIDS. The authors hold that the inconclusive evidence on the effectiveness of herbal medicines demand a degree of caution for its use. Also, because both western and herbal medicine have variable effectiveness, individuals should be allowed to access the preferred treatment option depending on the type of disease.

The credibility of the vendor of herbal medicine or herbal medicine practitioner/producer was of importance to participants [22, 23]. The trusted producer/vendor promotes herbal medicine use [8]. Therefore, herbal medicine vendors and practitioners should have adequate training to be informed in their practice. The environment where herbal medicine is sold should be clean and well structured. It was observed that vendors of herbal medicine and those who sold in cars are not credible because one may not trace these individuals if follow-up care is required or customers have concerns about the products [23]. Efforts should be made to ensure that herbal medicine is sold by qualified vendors. There is the need for policy development and enforcement to ensure that the production and sale of herbal medicine are done by qualified individuals. This will in the long run ensure the safety and efficacy of herbal drugs [18, 42]. There is a need for herbal medicine research to provide evidence based information to the general public to protect individuals from misuse of herbal medicine.

The cost of herbal medicine was considered high in this study and it was not covered by the National Health Insurance Scheme (NHIS). This assertion is of great concern because participants desire herbal medicine from credible sources and if the cost from the credible source is exorbitant, then it would lead to seeking services from unqualified people whose cost may be low [1]. It is anticipated that the NHIS will cover the cost of herbal medicine so that individuals who choose herbal medicine can access services from credible sources. In a lower middle income country such as Ghana, individuals face economic hardships and the NHIS could be very beneficial during illness. We anticipate that the NHIS could offer some relief to sick persons when it covers both essential western and herbal medicines. Accredited herbal medicine producers and vendors should make their products affordable so that all those who choose herbal medicine can afford.

## Conclusion

The use of herbal medicine to treat various health problems cannot be decoupled from contemporary health system. Freedom of choice of health care makes it imperative for health practitioners to realize that herbal medicine is an alternative treatment that patients use. The health care system should accommodate herbal medicine as an option of care to meet personal preferences of patients. Clients or patients who use herbal medicine should be educated on the product they use. Producers, vendors and prescribers of herbal medicine should make informed recommendations to users and prospective clients. Against the backdrop of all the negative perceptions of herbal medicine, people still use it. Therefore measures must be put in place to ensure that herbal products on the market are safe. To protect the general public from the use of harmful herbal products and ensure the quality of herbal medicine products, it is imperative to establish a government agency that regulates and control quality of herbal medicine products.

## Abbreviations

BSc: Bachelor of Science
CAM: complementary and alternative medicine
KNUST: Kwame Nkrumah University of Science and Technology
NHIS: National health insurance scheme
